# Supplementary material for: Real-World Study: Hybrid Immunity against SARS-CoV-2 Influences the Antibody Levels and Persistency Lasting More than One Year
Source: Vaccines (Basel). 2023 Nov 7;11(11):1693. doi: 10.3390/vaccines11111693 (PMC10674428; doi:10.3390/vaccines11111693)
Supplement: Supplementary file 1 [file vaccines-11-01693-s001.zip › vaccines-2676203-supplementary.pdf]

Supplementary information

Table S1. Binding antibody levels of participants who have received 2, 3, 4, or ≥ 5 doses, categorized by the duration since the last vaccination and vaccination plus infection.

	**(1)<br><6**	**(2)<br>6 to <9**	**(3)<br>9 to <12**	**(4)<br>≥12**
**N_{pos} groups**				
2 doses, n	2	2	7	16
GMT	24924.9	10615.3	1733.2	2642.7
[95%CI]			[506.0–5936.8]	[870.4–8023.3]
3 doses, n	9	17	32	43
GMT	10349.5	7142.2	5425.7	5965.7
[95%CI]	[5795.0–8483.7]	[4957.0–10290.7]	[4280.1–6877.9]	[4574.3–7780.4]
4 doses, n	31	62	91	74
GMT	8044.8	10483.7	6737.7	5847.9
[95%CI]	[5671.5–11411.3]	[8737.9–12578.2]	[5734.3–7916.7]	[4909.7–6965.4]
≥5 doses, n	38	52	72	5
GMT	15934.0	10988.4	8613.7	10361.8
[95%CI]	[13803.9–18392.9]	[8927.5–13525.0]	[7352.7–10090.9]	[4084.7–26285.7]
Overall, n	80	133	202	138
GMT	11778.4	10168.9	6779.6	5479.1
[95%CI]	[9900.8–14012.1]	[8970.9–11526.9]	[6066.5–7576.4]	[4590.4–6539.8]
**N_{neg} groups**				
2 doses, n	N/A	N/A	N/A	2
GMT				119.1
[95%CI]				
3 doses, n	N/A	1	5	27
GMT		1460.0	2820.9	1078.2
[95%CI]			[927.7–8577.8]	[791.9–1468.0]
4 doses, n	N/A	16	50	42
GMT		4175.2	2266.5	1889.3
[95%CI]		[2783.6–6262.4]	[1811.0–2836.5]	[1575.5–2265.5]
≥5 doses, n	8	30	35	2
GMT	10578.5	4433.2	3272.5	1184.5
[95%CI]	[5928.6–18875.4]	[3270.0–6010.1]	[2626.3–4077.8]	
Overall, n	8	47	90	73
GMT	10578.5	4242.2	2646.5	1405.3
[95%CI]	[5928.6–18875.4]	[3358.1–5359.0]	[2260.3–3098.7]	[1151.0–1715.7]

The duration of the last vaccination and vaccination plus infection is categorized as follows: (1) <6 months, (2) 6 to <9 months, (3) 9 to <12 months, and (4) ≥12 months.

Abbreviations: 95%CI, 95% confidence intervals; GMT, geometric mean titer in U/mL; n, number; N_{neg}, the seronegative of total anti-N Ig; N_{pos}, the seropositive of total anti-N Ig; N/A, no available.

HCW	n	45	53	94	75
	GMT	10933.5	8324.8	5621.6	3524.4
NHCW	n	43	127	198	136
	GMT	12480.6	7998.8	4832.0	3366.5

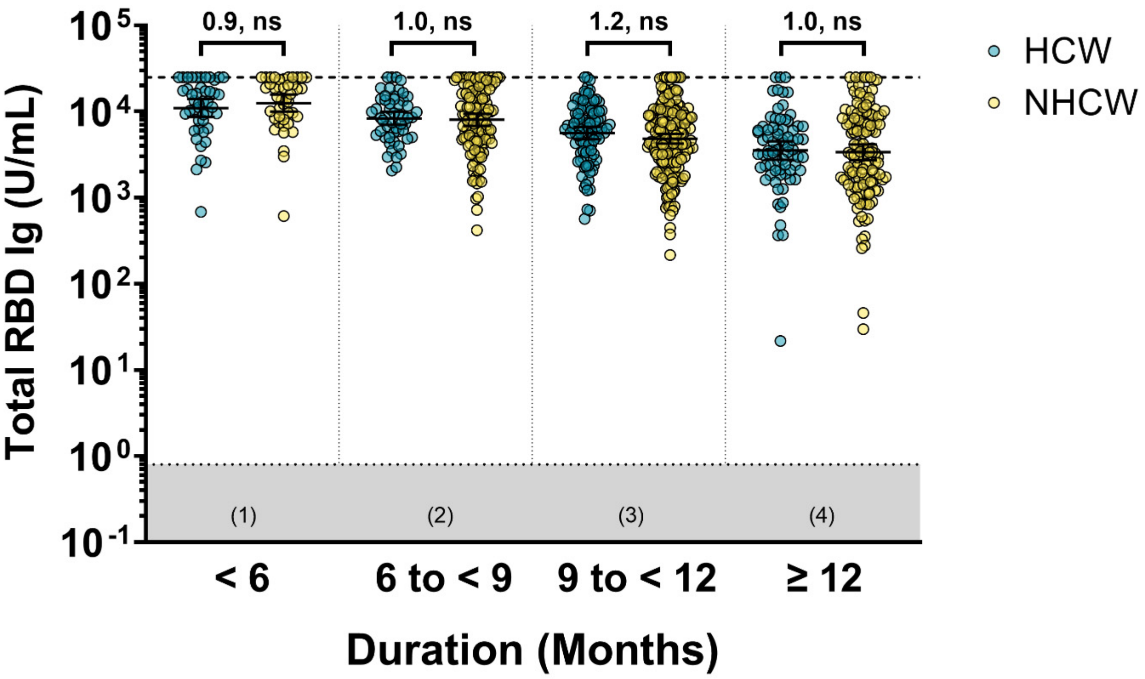

Figure S1. The total RBD Ig level of the participants was classified by the occupations (HCW and NHCW). The figure showed the relation between total RBD Ig (U/mL) and duration of last vaccination and vaccination plus infection, categorized as follows: (1) <6 months, (2) 6 to <9 months, (3) 9 to <12 months, and (4) ≥12 months. Lines represent the geometric mean titer (GMT) with 95% confidence intervals (95% CI). The upper limit of the total RBD Ig is reported as 25,000 U/mL. The gray area indicates the seronegativity of total RBD Ig (<0.8 U/mL). A pairwise comparison shows the geometric mean ratio (GMR) and statistical significance set at $p < 0.05$ (*), $p < 0.01$ (**), $p < 0.001$ (***), and no statistical significance (ns).
